# Supplementary material for: Sexual orientation-related disparities in employment, health insurance, healthcare access and health-related quality of life: a cohort study of US male and female adolescents and young adults
Source: BMJ Open. 2018 Jul 26;8(6):e020418. doi: 10.1136/bmjopen-2017-020418 (PMC6067349; doi:10.1136/bmjopen-2017-020418)
Supplement: Author's manuscript [file bmjopen-2017-020418.draft_revisions.pdf]

BMJ Open is committed to open peer review. As part of this commitment we make the peer review history of every article we publish publicly available.

When an article is published we post the peer reviewers' comments and the authors' responses online. We also post the versions of the paper that were used during peer review. These are the versions that the peer review comments apply to.

The versions of the paper that follow are the versions that were submitted during the peer review process. They are not the versions of record or the final published versions. They should not be cited or distributed as the published version of this manuscript.

BMJ Open is an open access journal and the full, final, typeset and author-corrected version of record of the manuscript is available on our site with no access controls, subscription charges or pay-per-view fees (http://bmjopen.bmj.com).

If you have any questions on BMJ Open's open peer review process please email info.bmjopen@bmj.com

BMJ Open

Sexual orientation-related disparities in employment, health insurance, healthcare access, and health-related quality of life: a longitudinal cohort of U.S. males and females

Journal:	*BMJ Open*
Manuscript ID	bmjopen-2017-020418
Article Type:	Research
Date Submitted by the Author:	09-Nov-2017
Complete List of Authors:	Charlton, Brittany; Harvard Medical School, ; Harvard University T H Chan School of Public Health,<br>Gordon, Allegra; Boston Children's Hospital<br>Reisner, Sari; Boston Children's Hospital<br>Sarda, Vishnudas; Boston Children's Hospital<br>Samnaliev, Mihail; Boston Children's Hospital<br>Austin, Bryn; Childrens Hospital Boston, Division of Adolescent and Young Adult Medicine
**Primary Subject Heading**:	Health economics
Secondary Subject Heading:	Epidemiology, General practice / Family practice, Health policy, Public health, Sexual health
Keywords:	EPIDEMIOLOGY, HEALTH ECONOMICS, HEALTH SERVICES ADMINISTRATION & MANAGEMENT, PUBLIC HEALTH, SEXUAL MEDICINE

SCHOLARONE™ Manuscripts

Title Page

Sexual orientation-related disparities in employment, health insurance, healthcare access, and health-related quality of life: a longitudinal cohort of U.S. males and females

Brittany M. Charlton[1-4], Allegra R. Gordon[1-2], Sari L. Reisner[2,4-6], Vishnudas Sarda[1], Mihail Samnaliev[1-2], S. Bryn Austin[1-3,7]

[1]Division of Adolescent/Young Adult Medicine, Boston Children's Hospital, Boston, USA
[2]Department of Pediatrics, Harvard Medical School, Boston, USA
[3]Channing Division of Network Medicine, Department of Medicine, Brigham and Women's Hospital, Boston, USA
[4]Department of Epidemiology, Harvard T.H. Chan School of Public Health, Boston, USA
[5]Division of General Pediatrics, Boston Children's Hospital, Boston, USA
[6]The Fenway Institute, Fenway Health, Boston, USA
[7]Department of Social and Behavioral Sciences, Harvard T.H. Chan School of Public Health, Boston, USA

Address correspondence to: Brittany M. Charlton, Division of Adolescent/Young Adult Medicine, Boston Children's Hospital, 300 Longwood Avenue, Boston, MA, USA. Telephone: (857) 218-5463 Fax: (617) 730-0004 Email address: bcharlton@mail.harvard.edu

Word count: 2,867

Keywords: Access to Health Care; Health Inequalities; Social Epidemiology; Quality of Life; Unemployment

For peer review only

ABSTRACT

Objectives: To investigate sexual orientation-related disparities in employment and healthcare including potential contributions to disparities in health-related quality of life (HRQL).

Setting: Growing Up Today Study, an ongoing a U.S.-based longitudinal cohort that began in 1996 and is primarily white and of middle-to-high socioeconomic positions.

Participants: 9,914 young adult participants aged 18-32 years of age at the most recent follow-up questionnaire.

Primary outcome measure: In 2013, participants reported if, in the last year, they had been unemployed, uninsured, or lacked healthcare access (a routine physical exam). Participants also completed the EQ-5D-5L, a validated, preference-weighted measurement of HRQL. After adjusting for potential confounders, we used sex-stratified, log-binomial models to calculate the association of sexual orientation with employment, health insurance, and healthcare access while also examining if these variables attenuated the sexual orientation-related HRQL disparities.

Results: Sexual minority females and males were about twice as likely as their respective heterosexual counterparts to have been unemployed and uninsured. Routine physical examination was not statistically different across sexual orientation groups. All sexual minority subgroups had worse HRQL than heterosexuals across each of the five EQ-5D-5L dimensions (mobility, self-care, usual activities, pain/discomfort, and anxiety/depression). Controlling for employment and health insurance did not substantially attenuate the existing sexual orientation-related HRQL disparities.

Conclusions: Previous research on sexual orientation-related disparities in employment and healthcare have often been limited to comparisons between cohabitating different- and same-sex adult couples, overlooking sexual minority subgroups (e.g., bisexuals versus lesbians), non-cohabitating populations, and young people. Less is known about sexual orientation-related disparities in HRQL including potential contributions from employment and healthcare. The current study documents that sexual orientation-related disparities in employment, health insurance, and various HRQL dimensions are pervasive across sexual minority subgroups, non-cohabitating couples, and young people even in U.S. families of middle-to-high socioeconomic positions.

ARTICLE SUMMARY: STRENGTHS AND LIMITATIONS OF THIS STUDY

- Cohort has predominantly high social status so findings may underestimate the prevalence of unemployment, a lack of health insurance, a lack of healthcare access, and poor HRQL
- There may be other factors that mediate the sexual orientation-related HRQL disparities including bullying victimization, social status, and others
- Data were cross-sectional and limited on some of our variables such as health insurance and healthcare access
- Includes a large sample drawn from young adults living across the United States
- This is the first study to examine these disparities across HRQL domains and evaluate the role of additional factors including employment, health insurance, and healthcare access

For peer review only

THUMBNAIL SKETCH

What is already known on this subject?

Compared to cohabitating couples in different-sex relationships, those in same-sex couples are more likely to be unemployed, lack health insurance, and lack healthcare access. However, we know little about other key populations such as those not cohabitating. Less is known about sexual orientation-related disparities in health-related quality of life including potential contributions from employment and healthcare.

What this study adds?

Sexual orientation-related disparities in employment and health insurance are pervasive even in U.S. families of middle-to-high socioeconomic positions. Until all people, regardless of sexual orientation, are treated equally in the eyes of the law with non-discrimination laws protecting employment as well as housing, public accommodations, and credit/lending, sexual orientation-related health disparities will persist.

For peer review only

For peer review only - http://bmjopen.bmj.com/site/about/guidelines.xhtml

INTRODUCTION

Half of all sexual minorities (e.g., lesbian, gay, and bisexual individuals) report employment discrimination in their lifetime[1]. Discrimination, along with other social and economic barriers, can lead to unemployment and, subsequently, a lack of health insurance and healthcare access. All of these factors may contribute to poor health-related quality of life (HRQL), which is a critical measure of health status. Comparing HRQL across different subpopulations can highlight disparities as well as help to evaluate the cost effectiveness of policies or programs that reduce such disparities[2].

Previous research has documented that, compared to heterosexuals, sexual minorities are more likely to: be unemployed, lack health insurance, and lack healthcare access[3–13]. However, these data are often limited to comparisons between cohabitating different-sex couples and same-sex adult couples, which precludes examination of differences in any other aspects such as among sexual minority subgroups (e.g., bisexuals versus lesbians), non-cohabitating groups, or young people. A few previous studies have also revealed that sexual minorities, on average, have worse quality of life than heterosexuals[14–19]. But, most of these data depend on limited measures of quality of life including measures that do not explicitly access HRQL, including its multiple dimensions, and few of these studies have examined potential mediators of the sexual orientation-related disparities in HRQL.

Documenting such disparities can provide policymakers with evidence to inform legislation that can lessen health inequities. For example, over half of states across the U.S. currently have no employment non-discrimination law covering sexual orientation[20] and documenting employment disparities, along with the potential downstream consequences—health insurance, healthcare access, and HRQL—can provide insight into the impact of policy changes. Therefore, the goal of this study was to leverage data from a U.S.-based longitudinal cohort of adolescents and young adults to examine sexual orientation-related disparities in employment, health insurance, and healthcare access while evaluating their contributions to HRQL disparities.

METHODS

Study Population

For the last 20 years, questionnaire data have been collected annually in the Growing Up Today Study (GUTS) from the female and male offspring of Nurses' Health Study 2 (NHS2) participants. The first wave of GUTS participants (GUTS1) was enrolled in 1996, when they were 9-14 years of age, and another wave of 9-16 year olds was enrolled in 2004 (GUTS2) making the entire cohort 19-31 years of age at the last questionnaire wave in 2013. We originally sent questionnaires to GUTS1 and 2 in alternating years but since 2013, we have combined GUTS1 and 2 into a single annual questionnaire. Participants' race/ethnicity is primarily white and most of their families report a middle-to-high household income (64% of participant's annual household income during their childhood was ≥$75,000).

The current analysis was limited to GUTS participants who reported their sexual orientation and information on the measures of unemployment, health insurance, healthcare access, and HRQL between baseline and the end of follow-up in 2013 (N=9,914). This study was approved by the Brigham and Women's Hospital Institutional Review Board.

Measures

Sexual orientation

Detailed information about sexual orientation has been repeatedly collected in GUTS 1 and 2 using an item adapted from the Minnesota Adolescent Health Survey[21], which asks about feelings of attraction and identity. The item read "Which of the following best describes your feelings?'' with the following response options: completely heterosexual (attracted to persons of the opposite sex), mostly heterosexual, bisexual (equally attracted to men and

For peer review only - http://bmjopen.bmj.com/site/about/guidelines.xhtml

women), mostly homosexual, completely homosexual (gay/lesbian, attracted to persons of the same sex), and not sure.

Sexual orientation groups were modeled using the 2013 questionnaire data (the same questionnaire year as the latest outcome) as follows: completely heterosexual (reference group), mostly heterosexual, bisexual, and lesbian/gay (made up from the mostly homosexual and completely homosexual groups). Missing data were imputed from previous questionnaire waves. Respondents endorsing “not sure” were excluded (n=69).

Unemployment

In 2010 (GUTS 1 only) and 2013, participants reported their employment status. Response options included: working full time, working part time, student, volunteering, military, unemployed/laid off/looking for work, staying at home with children/taking care of family, on maternity or family leave, and not working due to illness or disability. We categorized a participant as being unemployed/not working due to illness or disability in 2013 if they provided an affirmative response to the “unemployed/laid off/looking for work” item or the “not working due to illness or disability”.

Lacking health insurance

The 2013 questionnaire asked participants whether they were covered by any kind of health insurance or healthcare plan (yes; no). We categorized a participant as lacking health insurance in 2013 if they reported no such coverage.

Lacking healthcare access through routine physical exam

Use of routine physical exams was measured by asking about timing of last routine physical exam in 2013. We categorized participants who reported their last routine physical exam occurred >12 months before the questionnaire completion as not having healthcare access through a routine physical exam.

HRQL

The 2013 questionnaire assessed HRQL using the EQ-5D-5L[22], which is a validated, preference-weighted measure. EQ-5D-5L is a standardized, generic instrument that is applicable to a wide range of health conditions and appropriate for use with adolescents and young adults. This measure can also be used to calculate quality-adjusted life years (QALYs) for economic analyses with U.S.-based population weights.

EQ-5D-5L assesses HRQL in five dimensions (mobility, self-care, usual activities, pain/discomfort, anxiety/depression) by having participants report a dimension-specific score (1=no problems, 2=slight problems, 3=moderate problems, 4=severe problems, and 5=extreme problems). Based on the EuroQol Group recommendations[23], each physical functioning dimension was dichotomized into a score of 1 being “no problems” versus scores of 2 to 5 being “any problems.” The anxiety/depression dimension was dichotomized into scores of 1 to 2 being “none or slight problems” versus scores of 3 to 5 being “moderate, severe, or extreme problems,” as has been done previously[24].

All five dimensions were then used to create a summary HRQL index score[25]. Because EQ-5D-5L value sets are not yet available for the U.S., in order to preference-weight the index score for U.S. populations we relied on another value set to map EQ-5D-5L responses to the previous version, the EQ-5D-3L[26]. This summary results in an index score that is calibrated to reflect the degree to which different health statuses are valued in the U.S. population overall. Index scores for the U.S. population range from most severe impairment on all five dimensions, termed “worse than death” (value= -0.109), to full health (value=1.0)[27]. Previous research suggests that an index score difference as small as 0.02 points can have a

For peer review only - http://bmjopen.bmj.com/site/about/guidelines.xhtml

clinically meaningful difference[28]. We also analyzed the HRQL index score after dichotomizing it as a score of 1 being “full health” versus <1 being “not full health.”

Confounders

Potential confounders included baseline age in years, race/ethnicity (White, another race/ethnicity), childhood socioeconomic position (annual household income from NHS2 report in 2001 [<\$50,000; \$50,000-\$74,999; \$75,000-\$99,999; ≥\$100,000]), sex/gender, marital status in 2013 (married; not married), region of residence (West, Midwest, South, or Northeast), and cohort (GUTS 1, 2). If a participant’s data were missing for potential confounders, data were imputed from previous questionnaire years; if no such data were available for a participant then multiple imputation procedures were used.

Statistical Analysis

We first examined cross-sectional mean differences in employment, health insurance, healthcare access, and HRQL measures across sexual orientation groups. Multivariate regression from log-binomial models was used for dichotomous outcomes to calculate risk ratios (RR) and 95% confidence intervals (95%CI). Linear regression with the robust sandwich estimator was used for continuous outcomes to calculate betas (β) and standard errors. In order to account for sibling clusters, we estimated the variance using generalized estimating equations (GEE) with a compound symmetry working correlation matrix.

We calculated the risk ratios of experiencing unemployment, lacking health insurance, or lacking healthcare access by sexual orientation groups (referent=completely heterosexual), adjusted for potential confounders. Analyses for HRQL measures followed the previously used two-step approach[29,30] by first dichotomizing the index score (1 versus <1) and then using the continuous health index score in analyses restricted to those with lower HRQL (defined as health index scores <1). HRQL models were first adjusted for potential confounders and then adjusted for employment, health insurance, and healthcare access to explore attenuation. Previous research with this cohort[14] suggests possible effect modification of the sexual orientation and HRQL association by sex/gender but not by cohort so all analyses were stratified by sex/gender and adjusted for cohort. Analyses were conducted using SAS 9.3 (SAS Institute, Cary, NC).

RESULTS

Of the 9,914 participants in our sample, 7.5% were unemployed or not working due to illness or disability, 4.9% were uninsured, and 38.2% lacked healthcare access through a routine physical exam in the last year. As shown in Table 1, all of these outcomes varied by sexual orientation identity with sexual minorities having more unemployment, less health insurance, and less healthcare access (all *p*-values <0.01 except among men, where healthcare access was not statistically different across sexual orientation groups). The mean HRQL index score was 0.90 for females and 0.92 for males and varied by sexual orientation identity with sexual minorities having lower mean HRQL index scores compared to completely heterosexuals among both females and males (*p*-values <0.001). Within each of the five HRQL domains, sexual minority females and males were more likely than heterosexuals to report worse health (*p*-values ranged from <0.001 to 0.02 except among men, where self-care was not statistically different across sexual orientation groups).

For peer review only - http://bmjopen.bmj.com/site/about/guidelines.xhtml

Table 1. Age standardized characteristics of a cohort of U.S. females and males by sex/gender and sexual orientation (N=9,914).

Female (N=6,663)	Completely heterosexual (N=5,353)	Mostly heterosexual (N=1,037)	Bisexual (N=159)	Lesbian/Gay (N=114)	*p*-value[1]
Age at baseline[2], mean years (SD), Range: 18-32	26.1 (3.6)	26.6 (3.4)	25.5 (3.6)	26.4 (3.5)	<0.001
Unemployed/not working due to illness or disability in last year[3], % (N)	5.9 (317)	10.5 (109)	14.5 (23)	10.5 (12)	<0.001
Uninsured (lacked health insurance) in last year[3], % (N)	3.9 (208)	5.7 (59)	13.8 (22)	4.4 (5)	<0.001
Lacked a routine physical exam in the last year[3], % (N)	32.0 (1,712)	36.1 (374)	40.3 (64)	37.7 (43)	0.01
HRQL[4] dimensions, % (N)					
Mobility ≥slight problems	3.2 (172)	5.6 (58)	13.2 (21)	10.5 (12)	<0.001
Self-care ≥slight problems	0.5 (28)	1.3 (13)	4.4 (7)	1.8 (2)	<0.001
Usual activities ≥slight problems	5.0 (267)	12.3 (127)	20.1 (32)	16.7 (19)	<0.001
Pain/discomfort ≥slight problems	26.5 (1,418)	36.8 (382)	42.1 (67)	44.7 (51)	<0.001
Anxiety/depression ≥moderate problems	12.3 (658)	22.6 (234)	36.5 (58)	29.0 (33)	<0.001
HRQL index score[5], mean (SD)	0.91 (0.09)	0.87 (0.09)	0.84 (0.11)	0.85 (0.10)	<0.001
Less than full health (HRQL index score<1)	54.6 (2,924)	73.3 (760)	84.3 (134)	79.0 (90)	<0.001
HRQL index score among those with less than full health, mean (SD)	0.84 (0.06)	0.83 (0.06)	0.80 (0.09)	0.81 (0.08)	<0.001
**Male (N=3,251)**	(N=2,805)	(N=268)	(N=25)	(N=153)	
Age at baseline[2], mean years (SD), Range: 18-32	25.9 (3.7)	26.1 (3.7)	24.6 (3.8)	25.9 (3.6)	0.26
Unemployed/not working due to illness or disability in last year[3], % (N)	7.8 (218)	15.3 (41)	8.0 (2)	11.1 (17)	<0.001
Uninsured (lacked health insurance) in last year[3], % (N)	5.2 (147)	9.7 (26)	0.0 (0)	11.1 (17)	<0.001
Lacked a routine physical exam in the last year[3], % (N)	48.8 (1,369)	47.8 (128)	36.0 (9)	54.9 (84)	0.26
HRQL[4] dimensions, % (N)					
Mobility ≥slight problems	3.1 (86)	6.3 (17)	8.0 (2)	2.6 (4)	0.02
Self-care ≥slight problems	0.6 (18)	1.1 (3)	4.0 (1)	0.0 (0)	0.11
Usual activities ≥slight problems	3.8 (107)	10.5 (28)	8.0 (2)	5.9 (9)	<0.001
Pain/discomfort ≥slight problems	25.4 (711)	38.8 (104)	32.0 (8)	24.2 (37)	<0.001
Anxiety/depression ≥moderate problems	10.2 (285)	25.8 (69)	24.0 (6)	28.1 (43)	<0.001
HRQL index score[4], mean (SD)	0.92 (0.09)	0.87 (0.11)	0.86 (0.12)	0.89 (0.09)	<0.001
Less than full health (HRQL index score<1)	47.3 (1,329)	70.2 (188)	76.0 (19)	68.0 (104)	<0.001
HRQL index score among those with less than full health, mean (SD)	0.84 (0.06)	0.82 (0.08)	0.82 (0.11)	0.84 (0.06)	<0.001

[1]*p*-value calculated using ANOVA for continuous variables and chi square test for categorical variables (including those with a zero frequency cell such as the self-care HRQL dimension).
[2]Multiple imputation used in subsequent analyses for any missing covariates data. Percent missing: race/ethnicity (1.2%), marital status (0.2%), socioeconomic position (17.8%), and geographic region (0.1%).
[3]As reported in 2013.
[4]Health-related quality of life (HRQL) measured by the EQ-5D-5L, a validated preference-weighted measure for U.S. populations.
[5]Possible scores ranged from -0.109 (“worse than death”) to 1 (“full health”).

For peer review only

Table 2 presents the RRs and 95%CIs for the association between sexual orientation and the risk of being unemployed, lacking health insurance, or lacking healthcare access, adjusted for potential confounders. Compared to heterosexuals, sexual minority females and males were about twice as likely as their respective heterosexual counterparts to have been unemployed and uninsuranced. For example, bisexual females were at a higher risk than heterosexual females of being uninsuranced [RR (95%CI): 3.76 (2.42, 5.85)] and mostly heterosexual males were more likely than heterosexual males to have been unemployed [RR (95%CI): 1.82 (1.30, 2.54)]. Use of a routine physical exam was not statistically different across sexual orientation groups.

Table 2. Multivariable[1] risk ratios of experiencing unemployment or lacking health insurance or lacking healthcare access in a cohort of U.S. males and females by sex/gender and sexual orientation (N=9,914).

	Relative risk (95% CI)			
Female (N=6,663)	Completely heterosexual (N=5,353)	Mostly heterosexual (N=1,037)	Bisexual (N=159)	Lesbian/Gay (N=114)
Unemployed/not working due to illness or disability in last year[2]	1.00 (ref)	1.68 (1.35, 2.09)	2.39 (1.56, 3.65)	1.84 (1.03, 3.27)
Uninsured (lacked health insurance) in last year[2]	1.00 (ref)	1.39 (1.04, 1.86)	3.76 (2.42, 5.85)	1.18 (0.49, 2.88)
Lacked a routine physical exam in the last year[2]	1.00 (ref)	1.12 (1.00, 1.25)	1.26 (0.98, 1.62)	1.17 (0.86, 1.58)
Male (N=3,251)	(N=2,805)	(N=268)	(N=25)	(N=153)
Unemployed/not working due to illness or disability in last year[2]	1.00 (ref)	1.82 (1.30, 2.54)	1.00 (0.25, 4.06)	1.49 (0.91, 2.45)
Uninsured (lacked health insurance) in last year[2]	1.00 (ref)	1.67 (1.10, 2.54)	NA[3]	2.21 (1.33, 3.65)
Lacked a routine physical exam in the last year[2]	1.00 (ref)	0.97 (0.81, 1.16)	0.77 (0.40, 1.49)	1.13 (0.90, 1.40)

[1]Adjusted for age, race/ethnicity, childhood household income (reported by mother in 2001), geographic region, and cohort; multiple imputation used for missing covariates.
[2]As reported in 2013.
[3]Every bisexual male reported health insurance coverage in 2013 so no risk ratio was computed.

For peer review only

For peer review only - http://bmjopen.bmj.com/site/about/guidelines.xhtml

Table 3 provides the RRs and 95%CIs for the association between sexual orientation identity and the risk of having poorer HRQL (index score<1 versus 1). Model 0 demonstrates that, after accounting for potential confounders, sexual minority females and males had elevated risk of less-than-full health relative to completely heterosexuals. The addition of unemployment (model 1), lacking health insurance (model 2), lacking routine physical exam (model 3), or all three of these combined (model 4), did not substantially attenuate the associations between sexual orientation and HRQL.

Table 3. Multivariable[1] risk ratios of experiencing less than full health (HRQL index score<1 versus HRQL index score=1) in a cohort of U.S. males and females by sex/gender and sexual orientation (N=9,914).

	Relative risk (95% CI)			
Female (N=6,663)	Completely heterosexual (N=5353)	Mostly heterosexual (N=1,037)	Bisexual (N=159)	Lesbian/Gay (N=114)
Model 0: Sociodemographics	1.00 (ref)	1.33 (1.27, 1.39)	1.53 (1.42, 1.65)	1.42 (1.29, 1.57)
Model 1: Model 0 + unemployed	1.00 (ref)	1.32 (1.26, 1.38)	1.51 (1.40, 1.62)	1.41 (1.28, 1.56)
Model 2: Model 0 + uninsured	1.00 (ref)	1.32 (1.27, 1.38)	1.50 (1.39, 1.61)	1.42 (1.29, 1.57)
Model 3: Model 0 + lacked routine physical exam	1.00 (ref)	1.33 (1.27, 1.39)	1.53 (1.42, 1.65)	1.42 (1.29, 1.57)
Model 4: Model 0 + unemployed + uninsured + lacked routine physical exam	1.00 (ref)	1.32 (1.26, 1.38)	1.48 (1.37, 1.60)	1.41 (1.28, 1.56)
Male (N=3,251)	(N=2,805)	(N=268)	(N=25)	(N=153)
Model 0: Sociodemographics	1.00 (ref)	1.46 (1.34, 1.60)	1.62 (1.29, 2.03)	1.43 (1.27, 1.60)
Model 1: Model 0 + unemployed	1.00 (ref)	1.43 (1.31, 1.57)	1.62 (1.28, 2.05)	1.41 (1.26, 1.58)
Model 2: Model 0 + uninsured	1.00 (ref)	1.46 (1.33, 1.59)	1.63 (1.29, 2.04)	1.42 (1.26, 1.59)
Model 3: Model 0 + lacked routine physical exam	1.00 (ref)	1.46 (1.34, 1.60)	1.62 (1.29, 2.03)	1.43 (1.27, 1.60)
Model 4: Model 0 + unemployed + uninsured + lacked routine physical exam	1.00 (ref)	1.43 (1.31, 1.56)	1.62 (1.28, 2.05)	1.41 (1.26, 1.58)

[1]Adjusted for age, race/ethnicity, childhood household income (reported by mother in 2001), geographic region, and cohort; multiple imputation used for missing covariates.

For peer review only

For peer review only - http://bmjopen.bmj.com/site/about/guidelines.xhtml

Table 4 presents results for the multivariable linear regression of HRQL restricted to those with less-than-full health (index score<1). The patterns were similar to those provided in Table 3 with sexual minorities having lower HRQL compared to their completely heterosexual peers, and these relationships were not substantially attenuated after accounting for the effects of unemployment, lacking health insurance, and lacking a routine physical exam.

Table 4. Multivariable[1] linear associations between sexual orientation and HRQL index score in young adulthood among those who reported less than full health (score<1) in a cohort of U.S. males and females by sex/gender (N=5,547).

	β (standard error)					
Females (N=3,908)	Mostly heterosexual (N=760)	*p*-value	Bisexual (N=134)	*p*-value	Lesbian/Gay (N=90)	*p*-value
Model 0: Sociodemographics	-0.010 (0.003)	<.001	-0.034 (0.008)	<.001	-0.026 (0.008)	0.002
Model 1: Model 0 + unemployed	-0.010 (0.003)	<.001	-0.033 (0.008)	<.001	-0.025 (0.008)	0.002
Model 2: Model 0 + uninsured	-0.010 (0.003)	<.001	-0.033 (0.008)	<.001	-0.026 (0.008)	0.002
Model 3: Model 0 + lacked routine physical exam	-0.010 (0.003)	<.001	-0.034 (0.008)	<.001	-0.025 (0.008)	0.002
Model 4: Model 0 + unemployed + uninsured + lacked routine physical exam	-0.009 (0.003)	<.001	-0.033 (0.008)	<.001	-0.025 (0.008)	0.002
Males (N=1,639)	(N=188)		(N=19)		(N=104)	
Model 0: Sociodemographics	-0.020 (0.006)	0.001	-0.021 (0.025)	0.40	-0.004 (0.006)	0.47
Model 1: Model 0 + unemployed	-0.019 (0.006)	0.002	-0.022 (0.025)	0.38	-0.003 (0.006)	0.54
Model 2: Model 0 + uninsured	-0.020 (0.006)	0.001	-0.022 (0.025)	0.38	-0.004 (0.006)	0.54
Model 3: Model 0 + lacked routine physical exam	-0.020 (0.006)	0.001	-0.021 (0.025)	0.40	-0.004 (0.006)	0.47
Model 4: Model 0 + unemployed + uninsured + lacked routine physical exam	-0.019 (0.006)	0.002	-0.022 (0.025)	0.38	-0.003 (0.006)	0.58

[1]Adjusted for age, race/ethnicity, childhood household income (reported by mother in 2001), geographic region, and cohort; multiple imputation used for any missing covariates; completely heterosexual is the reference.

For peer review only - http://bmjopen.bmj.com/site/about/guidelines.xhtml

DISCUSSION

Sexual minority females and males are more likely than heterosexuals to have been unemployed and uninsuranced in early adulthood. Within each of the five HRQL dimensions, sexual minorities were also more likely than their heterosexual peers to report worse health. These disparities are pervasive even in a U.S. population that predominantly holds high social status with regards to race/ethnicity and socioeconomic position.

The existing literature on sexual orientation-related employment disparities primarily focuses on its contribution to more poverty among sexual minorities compared to heterosexuals[4–6,13]. Sexual minority women, as well as sexual minority people of color, appear to be especially vulnerable to poverty. Badgett et al. documented that policies to reduce wage gaps between heterosexual men and various sexual minority groups, including women and people of color, can significantly reduce poverty[5]. Although there is limited research on sexual orientation and employment status, data from the 2010 American Community Survey compare male same-sex couples and female same-sex couples to different-sex couples, all of which are cohabitating. These data reveal that cohabitating women in same-sex couples are more likely than women or men in cohabitating different-sex couples to be unemployed or not in the labor force. An estimated 40.4% of cohabitating women in same-sex couples were unemployed or not in the force compared to 21.8% of cohabitating men in same-sex relationships and 25.5% of cohabitating women and men different-sex couples. Women in same-sex couples benefit the least from employment as a way out of poverty because even among employed groups, women are at the highest risk of being in poverty[6]. The present study's findings support these data among sexual minority women. We also found larger employment disparities among sexual minority men possibly because our sample was not restricted to cohabitating participants.

Other studies have documented sexual orientation-related disparities in health insurance and healthcare access. For example, one of the largest nationally representative samples recently revealed that women in same-sex relationships were significantly less likely than women in different-sex relationships to have health insurance or to have had a check-up in the last year. Men in same-sex relationships in that sample were also less likely than men in different-sex relationships to have health insurance but more likely to have a check-up in the last year[7]. The present study supports most of the same patterns and a similar magnitude of health insurance and healthcare access disparities. A number of other studies document these same health insurance and healthcare access disparities using a cohabitating-based approach[3,9,11,12] and these results have also been repeated in other non-cohabitating samples as well[4,8,10]. However, many of these studies use samples that have limited statistical power, collapse sexual minority subgroups (e.g., lesbians and bisexuals), and are restricted to older adults who are in cohabitating relationships. The present study overcomes those challenges with a large sample allowing for improved statistical power and stratified sexual minority subgroups during late adolescent and early adulthood.

Our findings support the previous research that has identified lower HRQL among sexual minorities[14–19] using other quality of life measures[31–35]. Bisexual and lesbian women in the U.S.-based NHS2 cohort had lower HRQL scores than heterosexuals[15]. Certain sexual minority groups, including bisexual women and heterosexual men with same-sex partners, had lower HRQL scores than their heterosexual peers without same-sex partners in the state-based Representative California Quality of Life Survey[16]. Men who have sex with men in a Swedish sample had lower psychosocial HRQL scores than their heterosexual peers[17]. Other studies among college students have also identified lower quality of life among sexual minority females and male including one sample from Nigeria[18] and another from Cuba, Norway, India, and South Africa[19]. Another study from Washington State was restricted to sexual minorities, and therefore lacked a heterosexual comparison group; in this sample, young bisexual women had worse HRQL than their lesbian peers but these patterns reversed during midlife when lesbians

For peer review only - http://bmjopen.bmj.com/site/about/guidelines.xhtml

had worse HRQL[36]. No previous research, outside of the GUTS cohort[14], has used the EQ-5D-5L but the magnitude of our observed sexual orientation-related HRQL index scores differences is comparable or larger than those seen across individuals categorized by age, race/ethnicity, income, and education[27].

The GUTS cohort is made up of children of NHS2 participants, so results in this sample may not generalize to other populations; this cohort is predominantly White race/ethnicity, their mothers are all nurses, and the majority of participant's annual household income during their childhood was ≥$75,000. Given this high social status, our estimates may underestimate the prevalence of unemployment, a lack of health insurance, a lack of healthcare access, and poor HRQL. However, the fact that these disparities are pervasive in a cohort that predominantly holds high social status is striking. While our focus was on employment, health insurance, and healthcare access, there may be other factors that mediate the sexual orientation-related HRQL disparities including bullying victimization, social status, and others[14]. Data were cross-sectional and limited on some of our variables such as health insurance and healthcare access. Future longitudinal studies could explore more detailed types of health insurance coverage as well as other measurements of healthcare access.

This study has a number of strengths including the large sample drawn from young adults living across the United States. Building off the recently published data of sexual orientation-related disparities of HRQL index scores[14], this is the first study to examine these disparities across HRQL domains and evaluate the role of additional factors including employment, health insurance, and healthcare access. Utilizing the EQ-5D-5L to measure HRQL allows the findings to be incorporated into cost-effectiveness research, which can inform public policy decisions.

These sexual orientation-related disparities in employment and health insurance in a population with high social status highlight the ubiquity of sexual orientation inequities in the employment and healthcare systems. The U.S. Supreme Court's recent expansion of marriage rights to adults nationwide in same-sex relationships should lessen some of the sexual orientation-related disparities in health insurance. However, the adverse effects of previous bans are likely to persist[37,38]. Additionally, 28 states across the U.S. currently have no employment non-discrimination law covering sexual orientation—3 of these states have laws preventing the passage or enforcement of local non-discrimination laws[20]. Until all people, regardless of sexual orientation, are treated equally in the eyes of the law with non-discrimination laws protecting employment as well as housing, public accommodations, and credit/lending, sexual orientation-related health disparities will persist.

Contributorship: Dr. Charlton conceptualized the project, supervised the analyses, and led the development and writing of the article. Dr. Austin supervised data collection and, along with Drs. Gordon, Reisner, and Samnaliev, aided in the interpretation of data and critically reviewed the manuscript for important intellectual content. Mr. Sarda conducted the analyses.

Funding: Dr. Charlton was supported by grant F32HD084000 and Dr. Austin by R01HD057368 and R01HD066963 from the *Eunice Kennedy Shriver* National Institute of Child Health and Human Development, National Institutes of Health. Additional funds were provided to Dr. Charlton by GLMA: Health Professionals Advancing LGBT Equality's Lesbian Health Fund and by MRSG CPHPS 130006 from the American Cancer Society. Dr. Reisner was partly supported by grant CER-1403-12625 from the Patient-Centered Outcomes Research Institute. Additionally, an abstract of this work was presented at the Society for Adolescent Health and Medicine Annual Meeting and GLMA Annual Conference on LGBT Health.

Competing Interests: None declared.

For peer review only - http://bmjopen.bmj.com/site/about/guidelines.xhtml

Ethics Approval: This study was approved by the Brigham and Women's Hospital Institutional Review Board.

Data Sharing: No additional data available.

References

1 Sears B, Mallory C. Documented evidence of employment discrimination and its effects on LGBT people. The Williams Institute 2011.
2 Miller W, Robinson LA, Lawrence RS, editors. Valuing health for regulatory cost-effectiveness analysis. Washington, D.C.: : National Academies Press 2006. doi:10.17226/11534
3 Badgett MVL, Ash MA. Separate and unequal: the effect of unequal access to employment-based health insurance on gay, lesbian, and bisexual people. *Contemp Econ Policy* 2006;**24**:582–99.
4 Austin EL, Irwin JA. Health behaviors and health care utilization of southern lesbians. *Womens Heal Issues* 2010;**20**:178–84. doi:10.1016/j.whi.2010.01.002
5 Badgett MVL, Schneebaum A. The impact of wage equality on sexual orientation poverty gaps. The Williams Institute; 2015.
6 Badgett MVL, Durso LE, Schneebaum A. New patterns of poverty in the lesbian, gay, and bisexual community. The Williams Institute; 2013.
7 Buchmueller T, Carpenter CS. Disparities in Health Insurance Coverage, Access, and Outcomes for Individuals in Same-Sex Versus Different-Sex Relationships, 2000?2007. *Am J Public Health* 2010;**100**:489–95. doi:10.2105/AJPH.2009.160804
8 Diamant AL, Wold C, Spritzer K, *et al.* Health behaviors, health status, and access to and use of health care: a population-based study of lesbian, bisexual, and heterosexual women. *Arch Fam Med* 2000;**9**:1043–51.
9 Gonzales G, Blewett LA. National and state-specific health insurance disparities for adults in same-sex relationships. *Am J Public Health* 2014;**104**:e95–104. doi:10.2105/AJPH.2013.301577
10 Gonzales G, Henning-Smith C. The affordable care act and health insurance coverage for lesbian, gay, and bisexual adults: analysis of the behavioral risk factor surveillance system. *LGBT Heal* 2017;**4**:62–7. doi:10.1089/lgbt.2016.0023
11 Heck JE, Sell RL, Gorin SS. Health care access among individuals involved in same-sex relationships. *Am J Public Health* 2006;**96**:1111–8. doi:10.2105/AJPH.2005.062661
12 Pals H, Waren W. Winners and losers in health insurance: access and type of coverage for women in same-sex and opposite-sex partnerships. *Women Health* 2014;**54**:94–114. doi:10.1080/03630242.2013.870633
13 Prokos AH, Keene JR. Poverty among cohabiting gay and lesbian, and married and cohabiting heterosexual families. *J Fam Issues* 2010;**31**:934–59. doi:10.1177/0192513X09360176
14 Austin SB, Gordon AR, Ziyadeh NJ, *et al.* Stigma and health-related quality of life in sexual minorities. *Am J Prev Med* 2017;**57**:[Epub ahead of print] 566-587.
15 Case P, Austin SB, Hunter DJ, *et al.* Sexual orientation, health risk factors, and physical functioning in the Nurses' Health Study II. *J Womens Heal* 2004;**13**:1033–47. doi:10.1089/jwh.2004.13.1033
16 Cochran SD, Mays VM. Physical health complaints among lesbians, gay men, and bisexual and homosexually experienced heterosexual individuals: results from the California Quality of Life Survey. *Am J Public Health* 2007;**97**:2048–55. doi:10.2105/AJPH.2006.087254
17 Eriksson LE, Berglund T. Health-related quality of life in homo- and bisexual men

For peer review only - http://bmjopen.bmj.com/site/about/guidelines.xhtml

attending a sexually transmitted disease clinic in Sweden. *Int J STD AIDS* 2007;**18**:207–11. doi:10.1258/095646207780132442
18 Boladale M, Olakunle O, Olutayo A, *et al.* Sexual orientation and quality of life among students of Obafemi Awolowo University (OAU), Nigeria. *Afr Health Sci* 2015;**15**:1065–73. doi:10.4314/ahs.v15i4.3
19 Traeen B, Martinussen M, Vittersø J, *et al.* Sexual orientation and quality of life among university students from Cuba, Norway, India, and South Africa. *J Homosex* 2009;**56**:655–69. doi:10.1080/00918360903005311
20 Movement Advancement Project. Non-Discrimination Laws. 2017.http://www.lgbtmap.org/equality-maps/non_discrimination_laws (accessed 28 Jun 2017).
21 Remafedi G, Resnick M, Blum R, *et al.* Demography of sexual orientation in adolescents. *Pediatrics* 1992;**89**:714–21.
22 Johnson JA, Coons SJ. Comparison of the EQ-5D and SF-12 in an adult US sample. *Qual Life Res* 1998;**7**:155–66.
23 EuroQol Group. EQ-5D-5L. 2017.https://euroqol.org/eq-5d-instruments/eq-5d-5l-about/ (accessed 12 Jan 2017).
24 Gordon AR, Krieger N, Okechukwu CA, *et al.* Decrements in health-related quality of life associated with gender nonconformity among U.S. adolescents and young adults. Qual. Life Res. 2017;:1–10. doi:10.1007/s11136-017-1545-1
25 Agency for Healthcare Research and Quality. U.S. valuation of the EuroQol EQ-5 health states: research initiative in clinical economics. 2012.http://www.ahrq.gov/professionals/clinicians-providers/resources/rice/EQ5Dproj.html (accessed 12 Jan 2017).
26 van Hout B, Janssen MF, Feng Y-S, *et al.* Interim scoring for the EQ-5D-5L: mapping the EQ-5D-5L to EQ-5D-3L value sets. *Value Heal* 2012;**15**:708–15. doi:10.1016/j.jval.2012.02.008
27 Lubetkin EI, Jia H, Franks P, *et al.* Relationship among sociodemographic factors, clinical conditions, and health-related quality of life: examining the EQ-5D in the u.s. general population. *Qual Life Res* 2005;**14**:2187–96. doi:10.1007/s11136-005-8028-5
28 Goldsmith KA, Dyer MT, Schofield PM, *et al.* Relationship between the EQ-5D index and measures of clinical outcomes in selected studies of cardiovascular interventions. *Health Qual Life Outcomes* 2009;**7**:96. doi:10.1186/1477-7525-7-96
29 Pullenayegum EM, Tarride J-E, Xie F, *et al.* Analysis of health utility data when some subjects attain the upper bound of 1: are Tobit and CLAD models appropriate? *Value Heal* 2010;**13**:487–94. doi:10.1111/j.1524-4733.2010.00695.x
30 Bilger M, Finkelstein EA, Kruger E, *et al.* The effect of weight loss on health, productivity, and medical expenditures among overweight employees. *Med Care* 2013;**51**:471–7. doi:10.1097/MLR.0b013e318286e437
31 Pavot W, Diener E. Review of the satisfaction with life scale. *Psychol Assess* 1993;**5**:164–72. doi:10.1007/978-90-481-2354-4
32 Lyubomirsky S, Lepper HS. A measure of subjective happiness: preliminary reliability and construct validation. *Soc Indic Res* 1999;**46**:137–55. doi:10.1023/A:1006824100041
33 The World Health Organization Quality of Life Assessment (WHOQOL): development and general psychometric properties. *Soc Sci Med* 1998;**46**:1569–85.
34 Ware J, Kosinski M, Keller SD. A 12-item short-form health survey: construction of scales and preliminary tests of reliability and validity. *Med Care* 1996;**34**:220–33.
35 Ware J. *SF-36 physical and mental health summary scales: a user's manual*. Boston: : Health Institute New England Medical Center 1994.
36 Fredriksen-Goldsen KI, Kim H-J, Barkan SE, *et al.* Disparities in health-related quality of life: a comparison of lesbians and bisexual women. *Am J Public Health* 2010;**100**:2255–

For peer review only - http://bmjopen.bmj.com/site/about/guidelines.xhtml

61. doi:10.2105/AJPH.2009.177329

37 Hatzenbuehler ML, McLaughlin KA, Keyes KM, *et al.* The Impact of Institutional Discrimination on Psychiatric Disorders in Lesbian, Gay, and Bisexual Populations: A Prospective Study. *Am J Public Health* 2010;**100**:452–9. doi:10.2105/AJPH.2009.168815

38 Hatzenbuehler ML, O'Cleirigh C, Grasso C, *et al.* Effect of Same-Sex Marriage Laws on Health Care Use and Expenditures in Sexual Minority Men: A Quasi-Natural Experiment. *Am J Public Health* 2012;**102**:285–91. doi:10.2105/AJPH.2011.300382

For peer review only

STROBE Statement—Checklist of items that should be included in reports of ***cohort studies***

	Item No	Recommendation	Page
**Title and abstract**	1	(*a*) Indicate the study's design with a commonly used term in the title or the abstract	1
		(*b*) Provide in the abstract an informative and balanced summary of what was done and what was found	2
**Introduction**			
Background/rationale	2	Explain the scientific background and rationale for the investigation being reported	4
Objectives	3	State specific objectives, including any prespecified hypotheses	4
**Methods**			
Study design	4	Present key elements of study design early in the paper	4-6
Setting	5	Describe the setting, locations, and relevant dates, including periods of recruitment, exposure, follow-up, and data collection	4-6
Participants	6	(*a*) Give the eligibility criteria, and the sources and methods of selection of participants. Describe methods of follow-up	4-6
		(*b*) For matched studies, give matching criteria and number of exposed and unexposed	N/A
Variables	7	Clearly define all outcomes, exposures, predictors, potential confounders, and effect modifiers. Give diagnostic criteria, if applicable	4-6
Data sources/ measurement	8*	For each variable of interest, give sources of data and details of methods of assessment (measurement). Describe comparability of assessment methods if there is more than one group	4-6
Bias	9	Describe any efforts to address potential sources of bias	4-6
Study size	10	Explain how the study size was arrived at	4
Quantitative variables	11	Explain how quantitative variables were handled in the analyses. If applicable, describe which groupings were chosen and why	4-6
Statistical methods	12	(*a*) Describe all statistical methods, including those used to control for confounding	4-6
		(*b*) Describe any methods used to examine subgroups and interactions	4-6
		(*c*) Explain how missing data were addressed	4-6
		(*d*) If applicable, explain how loss to follow-up was addressed	4
		(*e*) Describe any sensitivity analyses	N/A
**Results**			
Participants	13*	(a) Report numbers of individuals at each stage of study—eg numbers potentially eligible, examined for eligibility, confirmed eligible, included in the study, completing follow-up, and analysed	6-10
		(b) Give reasons for non-participation at each stage	6-10
		(c) Consider use of a flow diagram	N/A
Descriptive data	14*	(a) Give characteristics of study participants (eg demographic, clinical, social) and information on exposures and potential confounders	6-7
		(b) Indicate number of participants with missing data for each variable of interest	5-6
		(c) Summarise follow-up time (eg, average and total amount)	4-6
Outcome data	15*	Report numbers of outcome events or summary measures over time	6-10
Main results	16	(*a*) Give unadjusted estimates and, if applicable, confounder-adjusted estimates and their precision (eg, 95% confidence interval). Make clear	6-10

For peer review only - http://bmjopen.bmj.com/site/about/guidelines.xhtml

		which confounders were adjusted for and why they were included	
		(*b*) Report category boundaries when continuous variables were categorized	6-10
		(*c*) If relevant, consider translating estimates of relative risk into absolute risk for a meaningful time period	N/A
Other analyses	17	Report other analyses done—eg analyses of subgroups and interactions, and sensitivity analyses	N/A
**Discussion**			
Key results	18	Summarise key results with reference to study objectives	11-12
Limitations	19	Discuss limitations of the study, taking into account sources of potential bias or imprecision. Discuss both direction and magnitude of any potential bias	11-12
Interpretation	20	Give a cautious overall interpretation of results considering objectives, limitations, multiplicity of analyses, results from similar studies, and other relevant evidence	12
Generalisability	21	Discuss the generalisability (external validity) of the study results	12
**Other information**			
Funding	22	Give the source of funding and the role of the funders for the present study and, if applicable, for the original study on which the present article is based	12

*Give information separately for exposed and unexposed groups.

Note: An Explanation and Elaboration article discusses each checklist item and gives methodological background and published examples of transparent reporting. The STROBE checklist is best used in conjunction with this article (freely available on the Web sites of PLoS Medicine at http://www.plosmedicine.org/, Annals of Internal Medicine at http://www.annals.org/, and Epidemiology at http://www.epidem.com/). Information on the STROBE Initiative is available at http://www.strobe-statement.org.

For peer review only

For peer review only - http://bmjopen.bmj.com/site/about/guidelines.xhtml

BMJ Open

Sexual orientation-related disparities in employment, health insurance, healthcare access, and health-related quality of life: a cohort study of U.S. male and female adolescents and young adults

Journal:	*BMJ Open*
Manuscript ID	bmjopen-2017-020418.R1
Article Type:	Research
Date Submitted by the Author:	27-Feb-2018
Complete List of Authors:	Charlton, Brittany; Harvard Medical School, ; Harvard University T H Chan School of Public Health,<br>Gordon, Allegra; Boston Children's Hospital<br>Reisner, Sari; Boston Children's Hospital<br>Sarda, Vishnudas; Boston Children's Hospital<br>Samnaliev, Mihail; Boston Children's Hospital<br>Austin, Bryn; Childrens Hospital Boston, Division of Adolescent and Young Adult Medicine
**Primary Subject Heading**:	Health economics
Secondary Subject Heading:	Epidemiology, General practice / Family practice, Health policy, Public health, Sexual health
Keywords:	EPIDEMIOLOGY, HEALTH ECONOMICS, HEALTH SERVICES ADMINISTRATION & MANAGEMENT, PUBLIC HEALTH, SEXUAL MEDICINE

SCHOLARONE™
Manuscripts

Title Page

Sexual orientation-related disparities in employment, health insurance, healthcare access, and health-related quality of life: a cohort study of U.S. male and female adolescents and young adults

Brittany M. Charlton[1-4], Allegra R. Gordon[1-2], Sari L. Reisner[2,4-6], Vishnudas Sarda[1], Mihail Samnaliev[1-2], S. Bryn Austin[1-3,7]

[1]Division of Adolescent/Young Adult Medicine, Boston Children's Hospital, Boston, USA
[2]Department of Pediatrics, Harvard Medical School, Boston, USA
[3]Channing Division of Network Medicine, Department of Medicine, Brigham and Women's Hospital, Boston, USA
[4]Department of Epidemiology, Harvard T.H. Chan School of Public Health, Boston, USA
[5]Division of General Pediatrics, Boston Children's Hospital, Boston, USA
[6]The Fenway Institute, Fenway Health, Boston, USA
[7]Department of Social and Behavioral Sciences, Harvard T.H. Chan School of Public Health, Boston, USA

Address correspondence to: Brittany M. Charlton, Division of Adolescent/Young Adult Medicine, Boston Children's Hospital, 300 Longwood Avenue, Boston, MA, USA. Telephone: (857) 218-5463 Fax: (617) 730-0004 Email address: bcharlton@mail.harvard.edu

Word count: 3,170

Keywords: Access to Health Care; Health Inequalities; Social Epidemiology; Quality of Life; Unemployment

For peer review only

ABSTRACT

Objectives: To investigate sexual orientation-related disparities in employment and healthcare including potential contributions from health-related quality of life (HRQL).

Setting: Growing Up Today Study, a U.S.-based longitudinal cohort begun in 1996; predominantly composed of participants who are white and of middle-to-high socioeconomic positions.

Participants: 9,914 participants 18-32 years old at the most recent follow-up questionnaire.

Primary outcome measure: In 2013, participants reported if, in the last year, they had been unemployed, uninsured, or lacked healthcare access (routine physical exam). Participants completed the EQ-5D-5L, a validated, preference-weighted measurement of HRQL. After adjusting for potential confounders, we used sex-stratified, log-binomial models to calculate the association of sexual orientation with employment, health insurance, and healthcare access while examining if these variables attenuated the sexual orientation-related HRQL disparities.

Results: Sexual minority females and males were about twice as likely as their respective heterosexual counterparts to have been unemployed and uninsured. For example, risk ratio (95% confidence interval) of uninsured bisexual females: 3.76 (2.42, 5.85) and unemployed mostly heterosexual males: 1.82 (1.30, 2.54). Routine physical examination was not different across sexual orientation groups ($p>0.05$). All sexual minority subgroups had worse HRQL than heterosexuals ($p<0.05$) across the five EQ-5D-5L dimensions (mobility, self-care, usual activities, pain/discomfort, and anxiety/depression). Controlling for employment and health insurance did not substantially attenuate the existing sexual orientation-related HRQL disparities.

Conclusions: Research on sexual orientation-related disparities in employment and healthcare has often been limited to comparisons between cohabitating different- and same-sex adult couples, overlooking sexual minority subgroups (e.g., bisexuals versus lesbians), non-cohabitating populations, and young people. Less is known about sexual orientation-related disparities in HRQL including potential contributions from employment and healthcare. The current study documents that disparities in employment, health insurance, and various HRQL dimensions are pervasive across sexual minority subgroups, non-cohabitating couples, and youth in families of middle-to-high socioeconomic positions.

ARTICLE SUMMARY: STRENGTHS AND LIMITATIONS OF THIS STUDY

- Cohort has predominantly high social status so while findings can examine sexual orientation-related differences, they may underestimate the prevalence of unemployment, a lack of health insurance, a lack of healthcare access, and poor HRQL compared to other populations (e.g., low social status, elderly)
- There may be other factors that mediate the sexual orientation-related HRQL disparities including bullying victimization, social status, and others
- Data were cross-sectional and limited on some of our variables such as health insurance and healthcare access
- Includes a large sample drawn from young adults living across the United States
- This is the first study to examine these disparities across HRQL domains and evaluate the role of additional factors including employment, health insurance, and healthcare access

For peer review only - http://bmjopen.bmj.com/site/about/guidelines.xhtml

INTRODUCTION

Nearly half of all sexual minorities (e.g., lesbian, gay, and bisexual individuals) report employment discrimination in their lifetime[1]. Discrimination, along with other social and economic barriers, can lead to unemployment and, subsequently, a lack of health insurance and healthcare access. All of these factors may contribute to poor health-related quality of life (HRQL), which is a critical measure of health status. Comparing HRQL across different subpopulations can highlight disparities as well as help to evaluate the cost effectiveness of policies or programs that reduce such disparities[2].

Previous research has documented that, compared to heterosexuals, sexual minorities are more likely to: be unemployed, lack health insurance, and lack healthcare access[3–13]. However, these data are often limited to comparisons between cohabitating different-sex couples and same-sex adult couples, which precludes examination of differences in any other aspects such as among sexual minority subgroups (e.g., bisexuals versus lesbians), non-cohabitating groups, or young people. A few previous studies have also revealed that sexual minorities, on average, have worse quality of life than heterosexuals[14–19]. But, most of these data depend on limited measures of quality of life including measures like the Medical Outcomes Study 36-Item Short-Form Health Survey (SF-36) which does not explicitly access HRQL, including its multiple dimensions (e.g., pain/discomfort, anxiety/depression). Additionally, few of these studies have examined potential mediators of the sexual orientation-related disparities in HRQL.

Documenting such disparities can provide policymakers with evidence to inform legislation that can lessen health inequities. For example, over half of states across the U.S. currently have no employment non-discrimination law covering sexual orientation[20] and documenting employment disparities. Using research to document the downstream consequences of unemployment—health insurance, healthcare access, and HRQL—can aid policy makers in crafting the necessary legal changes to lessen these inequities, such as federal employment non-discrimination laws. Therefore, the goal of this study was to leverage data from a U.S.-based longitudinal cohort of adolescents and young adults (ages 18-32) to examine sexual orientation-related disparities in employment, health insurance, and healthcare access while evaluating their contributions to HRQL disparities.

METHODS

Study Population

For the last 20 years, questionnaire data have been collected annually in the Growing Up Today Study (GUTS) from the female and male offspring of Nurses' Health Study 2 (NHS2) participants. The first wave of GUTS participants (GUTS1) was enrolled in 1996, when they were 9-14 years of age, and another wave of 9-16 year olds was enrolled in 2004 (GUTS2) making the entire cohort 18-32 years of age at the last questionnaire wave in 2013. We originally sent questionnaires to GUTS1 and 2 in alternating years but since 2013, we have combined GUTS1 and 2 into a single annual questionnaire. Participants' race/ethnicity is primarily white and most of their families report a middle-to-high household income (64% of participant's annual household income during their childhood was ≥$75,000).

The current analysis was limited to GUTS participants who reported their sexual orientation and information on the measures of unemployment, health insurance, healthcare access, and HRQL between baseline and the end of follow-up in 2013 (N=9,914). This study was approved by the Brigham and Women's Hospital Institutional Review Board.

Measures

Sexual orientation

Detailed information about sexual orientation has been repeatedly collected in GUTS 1 and 2 using an item adapted from the Minnesota Adolescent Health Survey[21], which asks

For peer review only - http://bmjopen.bmj.com/site/about/guidelines.xhtml

about feelings of attraction and identity. The item read "Which of the following best describes your feelings?'' with the following response options: completely heterosexual (attracted to persons of the opposite sex), mostly heterosexual, bisexual (equally attracted to men and women), mostly homosexual, completely homosexual (gay/lesbian, attracted to persons of the same sex), and not sure.

Sexual orientation groups were modeled using the 2013 questionnaire data (the same questionnaire year as the latest outcome) as follows: completely heterosexual (reference group), mostly heterosexual, bisexual, and lesbian/gay (made up from the mostly homosexual and completely homosexual groups). Missing data were limited and imputed from previous questionnaire waves. Respondents endorsing "not sure" were excluded (n=69).

Unemployment

In 2010 (GUTS 1 only) and 2013, participants reported their employment status. Response options included: working full time, working part time, student, volunteering, military, unemployed/laid off/looking for work, staying at home with children/taking care of family, on maternity or family leave, and not working due to illness or disability. We categorized a participant as being unemployed/not working due to illness or disability in 2013 if they provided an affirmative response to the "unemployed/laid off/looking for work" item or the "not working due to illness or disability".

Lacking health insurance

The 2013 questionnaire asked participants whether they were covered by any kind of health insurance or healthcare plan (yes; no). We categorized a participant as lacking health insurance in 2013 if they reported no such coverage.

Lacking healthcare access through routine physical exam

Use of routine physical exams was measured by asking about timing of last routine physical exam in 2013. We categorized participants who reported their last routine physical exam occurred >12 months before the questionnaire completion as not having healthcare access through a routine physical exam.

HRQL

The 2013 questionnaire assessed HRQL using the EQ-5D-5L[22], which is a validated, preference-weighted measure. EQ-5D-5L is a standardized, generic instrument that is applicable to a wide range of health conditions and appropriate for use with adolescents and young adults. This measure can also be used to calculate quality-adjusted life years (QALYs) for economic analyses with U.S.-based population weights.

EQ-5D-5L assesses HRQL in five dimensions (mobility, self-care, usual activities, pain/discomfort, anxiety/depression) by having participants report a dimension-specific score (1=no problems, 2=slight problems, 3=moderate problems, 4=severe problems, and 5=extreme problems). Based on the EuroQol Group recommendations[23], each physical functioning dimension was dichotomized into a score of 1 being "no problems" versus scores of 2 to 5 being "any problems." The anxiety/depression dimension was dichotomized into scores of 1 to 2 being "none or slight problems" versus scores of 3 to 5 being "moderate, severe, or extreme problems," as has been done previously[24].

All five dimensions were then used to create a summary HRQL index score[25]. Because EQ-5D-5L value sets are not yet available for the U.S., in order to preference-weight the index score for U.S. populations we relied on another value set to map EQ-5D-5L responses to the previous version, the EQ-5D-3L[26]. This summary results in an index score that is calibrated to reflect the degree to which different health statuses are valued in the U.S. population overall. Index scores for the U.S. population range from most severe impairment on

For peer review only - http://bmjopen.bmj.com/site/about/guidelines.xhtml

all five dimensions, termed “worse than death” (value= -0.109), to full health (value=1.0)[27]. Previous research suggests that an index score difference as small as 0.02 points can have a clinically meaningful difference[28]. We also analyzed the HRQL index score after dichotomizing it as a score of 1 being “full health” versus <1 being “not full health.”

Confounders

Potential confounders included baseline age in years, race/ethnicity (White, another race/ethnicity), childhood socioeconomic position (annual household income from NHS2 report in 2001 [<$50,000; $50,000-$74,999; $75,000-$99,999; ≥$100,000]), sex/gender, marital status in 2013 (married; not married), region of residence (West, Midwest, South, or Northeast), and cohort (GUTS 1, 2). If a participant’s data were missing for potential confounders, data were imputed from previous questionnaire years; if no such data were available for a participant then multiple imputation procedures were used.

Statistical Analysis

We first examined cross-sectional mean differences in employment, health insurance, healthcare access, and HRQL measures across sexual orientation groups. Multivariate regression from log-binomial models was used for dichotomous outcomes to calculate risk ratios (RR) and 95% confidence intervals (95%CI). Linear regression with the robust sandwich estimator was used for continuous outcomes to calculate betas (β) and standard errors. In order to account for sibling clusters, we estimated the variance using generalized estimating equations (GEE) with a compound symmetry working correlation matrix.

We calculated the risk ratios of experiencing unemployment, lacking health insurance, or lacking healthcare access by sexual orientation groups (referent=completely heterosexual), adjusted for potential confounders. Analyses for HRQL measures followed the previously used two-step approach[29,30] by first dichotomizing the index score (1 versus <1) and then using the continuous health index score in analyses restricted to those with lower HRQL (defined as health index scores <1). HRQL models were first adjusted for potential confounders and then adjusted for employment, health insurance, and healthcare access to explore attenuation. Previous research with this cohort[14] suggests possible effect modification of the sexual orientation and HRQL association by sex/gender but not by cohort so all analyses were stratified by sex/gender and adjusted for cohort. Analyses were conducted using SAS 9.3 (SAS Institute, Cary, NC).

Patient and Public Involvement

The public, including patients and study participants, were not involved in setting the research question or the outcome measures, nor was the public involved in developing plans for the study’s design, recruitment, or implementation. GUTS research results are regularly reported to study participants including through newsletters and other communications.

RESULTS

Of the 9,914 participants in our sample, 7.5% were unemployed or not working due to illness or disability, 4.9% were uninsured, and 38.2% lacked healthcare access through a routine physical exam in the last year. As shown in Table 1, all of these outcomes varied by sexual orientation identity with sexual minorities having more unemployment, less health insurance, and less healthcare access (all *p*-values <0.01 except among men, where healthcare access was not statistically different across sexual orientation groups). The mean HRQL index score was 0.90 for females and 0.92 for males and varied by sexual orientation identity with sexual minorities having lower mean HRQL index scores compared to completely heterosexuals among both females and males (*p*-values <0.001). Within each of the five HRQL domains, sexual minority females and males were more likely than heterosexuals to report worse health

For peer review only - http://bmjopen.bmj.com/site/about/guidelines.xhtml

(*p*-values ranged from <0.001 to 0.02 except among men, where self-care was not statistically different across sexual orientation groups).

For peer review only

For peer review only - http://bmjopen.bmj.com/site/about/guidelines.xhtml

Table 1. Age standardized characteristics of a cohort of U.S. females and males by sex/gender and sexual orientation (N=9,914).

Female (N=6,663)	Completely heterosexual (N=5,353)	Mostly heterosexual (N=1,037)	Bisexual (N=159)	Lesbian/Gay (N=114)	*p*-value[1]
Age at baseline[2], mean years (SD), Range: 18-32	26.1 (3.6)	26.6 (3.4)	25.5 (3.6)	26.4 (3.5)	<0.001
Unemployed/not working due to illness or disability in last year[3], % (N)	5.9 (317)	10.5 (109)	14.5 (23)	10.5 (12)	<0.001
Uninsured (lacked health insurance) in last year[3], % (N)	3.9 (208)	5.7 (59)	13.8 (22)	4.4 (5)	<0.001
Lacked a routine physical exam in the last year[3], % (N)	32.0 (1,712)	36.1 (374)	40.3 (64)	37.7 (43)	0.01
HRQL[4] dimensions, % (N)					
Mobility ≥slight problems	3.2 (172)	5.6 (58)	13.2 (21)	10.5 (12)	<0.001
Self-care ≥slight problems	0.5 (28)	1.3 (13)	4.4 (7)	1.8 (2)	<0.001
Usual activities ≥slight problems	5.0 (267)	12.3 (127)	20.1 (32)	16.7 (19)	<0.001
Pain/discomfort ≥slight problems	26.5 (1,418)	36.8 (382)	42.1 (67)	44.7 (51)	<0.001
Anxiety/depression ≥moderate problems	12.3 (658)	22.6 (234)	36.5 (58)	29.0 (33)	<0.001
HRQL index score[5], mean (SD)	0.91 (0.09)	0.87 (0.09)	0.84 (0.11)	0.85 (0.10)	<0.001
Less than full health (HRQL index score<1)	54.6 (2,924)	73.3 (760)	84.3 (134)	79.0 (90)	<0.001
HRQL index score among those with less than full health, mean (SD)	0.84 (0.06)	0.83 (0.06)	0.80 (0.09)	0.81 (0.08)	<0.001
Male (N=3,251)	(N=2,805)	(N=268)	(N=25)	(N=153)	
Age at baseline[2], mean years (SD), Range: 18-32	25.9 (3.7)	26.1 (3.7)	24.6 (3.8)	25.9 (3.6)	0.26
Unemployed/not working due to illness or disability in last year[3], % (N)	7.8 (218)	15.3 (41)	8.0 (2)	11.1 (17)	<0.001
Uninsured (lacked health insurance) in last year[3], % (N)	5.2 (147)	9.7 (26)	0.0 (0)	11.1 (17)	<0.001
Lacked a routine physical exam in the last year[3], % (N)	48.8 (1,369)	47.8 (128)	36.0 (9)	54.9 (84)	0.26
HRQL[4] dimensions, % (N)					
Mobility ≥slight problems	3.1 (86)	6.3 (17)	8.0 (2)	2.6 (4)	0.02
Self-care ≥slight problems	0.6 (18)	1.1 (3)	4.0 (1)	0.0 (0)	0.11
Usual activities ≥slight problems	3.8 (107)	10.5 (28)	8.0 (2)	5.9 (9)	<0.001
Pain/discomfort ≥slight problems	25.4 (711)	38.8 (104)	32.0 (8)	24.2 (37)	<0.001
Anxiety/depression ≥moderate problems	10.2 (285)	25.8 (69)	24.0 (6)	28.1 (43)	<0.001
HRQL index score[4], mean (SD)	0.92 (0.09)	0.87 (0.11)	0.86 (0.12)	0.89 (0.09)	<0.001
Less than full health (HRQL index score<1)	47.3 (1,329)	70.2 (188)	76.0 (19)	68.0 (104)	<0.001
HRQL index score among those with less than full health, mean (SD)	0.84 (0.06)	0.82 (0.08)	0.82 (0.11)	0.84 (0.06)	<0.001

[1]*p*-value calculated using ANOVA for continuous variables and chi square test for categorical variables (including those with a zero frequency cell such as the self-care HRQL dimension).
[2]Multiple imputation used in subsequent analyses for any missing covariates data. Percent missing: race/ethnicity (1.2%), marital status (0.2%), socioeconomic position (17.8%), and geographic region (0.1%).
[3]As reported in 2013.
[4]Health-related quality of life (HRQL) measured by the EQ-5D-5L, a validated preference-weighted measure for U.S. populations.
[5]Possible scores ranged from -0.109 (“worse than death”) to 1 (“full health”).

For peer review only - http://bmjopen.bmj.com/site/about/guidelines.xhtml

Table 2 presents the RRs and 95%CIs for the association between sexual orientation and the risk of being unemployed, lacking health insurance, or lacking healthcare access, adjusted for potential confounders. Compared to heterosexuals, sexual minority females and males were about twice as likely as their respective heterosexual counterparts to have been unemployed and uninsuranced. For example, bisexual females were at a higher risk than heterosexual females of being uninsuranced [RR (95%CI): 3.76 (2.42, 5.85)] and mostly heterosexual males were more likely than heterosexual males to have been unemployed [RR (95%CI): 1.82 (1.30, 2.54)]. Use of a routine physical exam was not statistically different across sexual orientation groups.

Table 2. Multivariable[1] risk ratios of experiencing unemployment or lacking health insurance or lacking healthcare access in a cohort of U.S. males and females by sex/gender and sexual orientation (N=9,914).

	Relative risk (95% CI)			
Female (N=6,663)	Completely heterosexual (N=5,353)	Mostly heterosexual (N=1,037)	Bisexual (N=159)	Lesbian/Gay (N=114)
Unemployed/not working due to illness or disability in last year[2]	1.00 (ref)	1.68 (1.35, 2.09)	2.39 (1.56, 3.65)	1.84 (1.03, 3.27)
Uninsured (lacked health insurance) in last year[2]	1.00 (ref)	1.39 (1.04, 1.86)	3.76 (2.42, 5.85)	1.18 (0.49, 2.88)
Lacked a routine physical exam in the last year[2]	1.00 (ref)	1.12 (1.00, 1.25)	1.26 (0.98, 1.62)	1.17 (0.86, 1.58)
Male (N=3,251)	(N=2,805)	(N=268)	(N=25)	(N=153)
Unemployed/not working due to illness or disability in last year[2]	1.00 (ref)	1.82 (1.30, 2.54)	1.00 (0.25, 4.06)	1.49 (0.91, 2.45)
Uninsured (lacked health insurance) in last year[2]	1.00 (ref)	1.67 (1.10, 2.54)	NA[3]	2.21 (1.33, 3.65)
Lacked a routine physical exam in the last year[2]	1.00 (ref)	0.97 (0.81, 1.16)	0.77 (0.40, 1.49)	1.13 (0.90, 1.40)

[1]Adjusted for age, race/ethnicity, childhood household income (reported by mother in 2001), geographic region, and cohort; multiple imputation used for missing covariates.
[2]As reported in 2013.
[3]Every bisexual male reported health insurance coverage in 2013 so no risk ratio was computed.

For peer review only

For peer review only - http://bmjopen.bmj.com/site/about/guidelines.xhtml

Table 3 provides the RRs and 95%CIs for the association between sexual orientation identity and the risk of having poorer HRQL (index score<1 versus 1). Model 0 demonstrates that, after accounting for potential confounders, sexual minority females and males had elevated risk of less-than-full health relative to completely heterosexuals. The addition of unemployment (model 1), lacking health insurance (model 2), lacking routine physical exam (model 3), or all three of these combined (model 4), did not substantially attenuate the associations between sexual orientation and HRQL.

Table 3. Multivariable[1] risk ratios of experiencing less than full health (HRQL index score<1 versus HRQL index score=1) in a cohort of U.S. males and females by sex/gender and sexual orientation (N=9,914).

	Relative risk (95% CI)			
Female (N=6,663)	Completely heterosexual (N=5353)	Mostly heterosexual (N=1,037)	Bisexual (N=159)	Lesbian/Gay (N=114)
Model 0: Sociodemographics	1.00 (ref)	1.33 (1.27, 1.39)	1.53 (1.42, 1.65)	1.42 (1.29, 1.57)
Model 1: Model 0 + unemployed	1.00 (ref)	1.32 (1.26, 1.38)	1.51 (1.40, 1.62)	1.41 (1.28, 1.56)
Model 2: Model 0 + uninsured	1.00 (ref)	1.32 (1.27, 1.38)	1.50 (1.39, 1.61)	1.42 (1.29, 1.57)
Model 3: Model 0 + lacked routine physical exam	1.00 (ref)	1.33 (1.27, 1.39)	1.53 (1.42, 1.65)	1.42 (1.29, 1.57)
Model 4: Model 0 + unemployed + uninsured + lacked routine physical exam	1.00 (ref)	1.32 (1.26, 1.38)	1.48 (1.37, 1.60)	1.41 (1.28, 1.56)
Male (N=3,251)	(N=2,805)	(N=268)	(N=25)	(N=153)
Model 0: Sociodemographics	1.00 (ref)	1.46 (1.34, 1.60)	1.62 (1.29, 2.03)	1.43 (1.27, 1.60)
Model 1: Model 0 + unemployed	1.00 (ref)	1.43 (1.31, 1.57)	1.62 (1.28, 2.05)	1.41 (1.26, 1.58)
Model 2: Model 0 + uninsured	1.00 (ref)	1.46 (1.33, 1.59)	1.63 (1.29, 2.04)	1.42 (1.26, 1.59)
Model 3: Model 0 + lacked routine physical exam	1.00 (ref)	1.46 (1.34, 1.60)	1.62 (1.29, 2.03)	1.43 (1.27, 1.60)
Model 4: Model 0 + unemployed + uninsured + lacked routine physical exam	1.00 (ref)	1.43 (1.31, 1.56)	1.62 (1.28, 2.05)	1.41 (1.26, 1.58)

[1]Adjusted for age, race/ethnicity, childhood household income (reported by mother in 2001), geographic region, and cohort; multiple imputation used for missing covariates.

For peer review only

Table 4 presents results for the multivariable linear regression of HRQL restricted to those with less-than-full health (index score<1). The patterns were similar to those provided in Table 3 with sexual minorities having lower HRQL compared to their completely heterosexual peers, and these relationships were not substantially attenuated after accounting for the effects of unemployment, lacking health insurance, and lacking a routine physical exam.

Table 4. Multivariable[1] linear associations between sexual orientation and HRQL index score in young adulthood among those who reported less than full health (score<1) in a cohort of U.S. males and females by sex/gender (N=5,547).

	β (standard error)					
Females (N=3,908)	Mostly heterosexual (N=760)	*p*-value	Bisexual (N=134)	*p*-value	Lesbian/Gay (N=90)	*p*-value
Model 0: Sociodemographics	-0.010 (0.003)	<.001	-0.034 (0.008)	<.001	-0.026 (0.008)	0.002
Model 1: Model 0 + unemployed	-0.010 (0.003)	<.001	-0.033 (0.008)	<.001	-0.025 (0.008)	0.002
Model 2: Model 0 + uninsured	-0.010 (0.003)	<.001	-0.033 (0.008)	<.001	-0.026 (0.008)	0.002
Model 3: Model 0 + lacked routine physical exam	-0.010 (0.003)	<.001	-0.034 (0.008)	<.001	-0.025 (0.008)	0.002
Model 4: Model 0 + unemployed + uninsured + lacked routine physical exam	-0.009 (0.003)	<.001	-0.033 (0.008)	<.001	-0.025 (0.008)	0.002
Males (N=1,639)	(N=188)		(N=19)		(N=104)	
Model 0: Sociodemographics	-0.020 (0.006)	0.001	-0.021 (0.025)	0.40	-0.004 (0.006)	0.47
Model 1: Model 0 + unemployed	-0.019 (0.006)	0.002	-0.022 (0.025)	0.38	-0.003 (0.006)	0.54
Model 2: Model 0 + uninsured	-0.020 (0.006)	0.001	-0.022 (0.025)	0.38	-0.004 (0.006)	0.54
Model 3: Model 0 + lacked routine physical exam	-0.020 (0.006)	0.001	-0.021 (0.025)	0.40	-0.004 (0.006)	0.47
Model 4: Model 0 + unemployed + uninsured + lacked routine physical exam	-0.019 (0.006)	0.002	-0.022 (0.025)	0.38	-0.003 (0.006)	0.58

[1]Adjusted for age, race/ethnicity, childhood household income (reported by mother in 2001), geographic region, and cohort; multiple imputation used for any missing covariates; completely heterosexual is the reference.

For peer review only

DISCUSSION

Sexual minority females and males are more likely than heterosexuals to have been unemployed and uninsured in early adulthood. Within each of the five HRQL dimensions, sexual minorities were also more likely than their heterosexual peers to report worse health. These disparities are pervasive in a U.S. population that predominantly holds high social status with regards to race/ethnicity and socioeconomic position.

The existing literature on sexual orientation-related employment disparities primarily focuses on its contribution to more poverty among sexual minorities compared to heterosexuals[4–6,13]. Sexual minority women, as well as sexual minority people of color, appear to be especially vulnerable to poverty. Badgett et al. documented that policies to reduce wage gaps between heterosexual men and various sexual minority groups, including women and people of color, can significantly reduce poverty[5]. Although there is limited research on sexual orientation and employment status, data from the 2010 American Community Survey compare male same-sex couples and female same-sex couples to different-sex couples, all of which are cohabitating. These data reveal that cohabitating women in same-sex couples are more likely than women or men in cohabitating different-sex couples to be unemployed or not in the labor force. An estimated 40.4% of cohabitating women in same-sex couples were unemployed or not in the work force compared to 21.8% of cohabitating men in same-sex relationships and 25.5% of cohabitating women and men different-sex couples. Women in same-sex couples benefit the least from employment as a way out of poverty because even among employed groups, women are at the highest risk of being in poverty[6]. The present study's findings support these data revealing higher unemployment among sexual minority women compared to heterosexual women. We also found larger employment disparities among sexual minority men possibly because our sample was not restricted to cohabitating participants.

Other studies have documented sexual orientation-related disparities in health insurance and healthcare access. For example, one of the largest nationally representative samples recently revealed that women in same-sex relationships were significantly less likely than women in different-sex relationships to have health insurance or to have had a check-up in the last year. Men in same-sex relationships in that sample were also less likely than men in different-sex relationships to have health insurance but more likely to have a check-up in the last year[7]. The present study supports most of the same patterns and a similar magnitude of health insurance and healthcare access disparities. A number of other studies document these same health insurance and healthcare access disparities using a cohabitating-based approach[3,9,11,12] and these results have also been repeated in other non-cohabitating samples as well[4,8,10]. However, many of these studies use samples that have limited statistical power, collapse sexual minority subgroups (e.g., lesbians and bisexuals), and are restricted to older adults who are in cohabitating relationships. The present study overcomes those challenges with a large sample allowing for improved statistical power and stratified sexual minority subgroups during late adolescent and early adulthood.

Our findings support the previous research that has identified lower HRQL among sexual minorities[14–19] using other quality of life measures[31–35]. Bisexual and lesbian women in the U.S.-based NHS2 cohort had lower HRQL scores than heterosexuals[15]. Certain sexual minority groups, including bisexual women and heterosexual men with same-sex partners, had lower HRQL scores than their heterosexual peers without same-sex partners in the state-based Representative California Quality of Life Survey[16]. Men who have sex with men in a Swedish sample had lower psychosocial HRQL scores than their heterosexual peers[17]. Other studies among college students have also identified lower quality of life among sexual minority females and male including one sample from Nigeria[18] and another from Cuba, Norway, India, and South Africa[19]. Another study from Washington State was restricted to sexual minorities, and therefore lacked a heterosexual comparison group; in this sample, young bisexual women had

For peer review only - http://bmjopen.bmj.com/site/about/guidelines.xhtml

worse HRQL than their lesbian peers but these patterns reversed during midlife when lesbians had worse HRQL[36]. Research on sexual orientation-related HRQL disparities using the EQ-5D-5L is scare but this measure was used in two recent publications, one from the GUTS cohort[14] and another based on a sample in Barcelona, Spain[37]. The current findings support those two publications findings that sexual minorities participants presented worse HRQL than heterosexuals while the current study also adds new insights. For example, the previous GUTS publication did not include estimates of the different HRQL dimensions—nor the contribution from employment and healthcare—and the Spanish sample was not large enough to examine sexual minority subgroups.

In addition to the differences observed in the current study comparing sexual minorities to heterosexuals, there were also notable differences comparing males and females as well as across sexual orientation subgroups. Male participants were more likely than females to have lacked a physical exam within the last year but less likely than females to have experienced pain/discomfort (one of the five HRQL dimensions). These patterns align with existing literature on men being less likely than women to seek healthcare[38] and the prevalence of pain that women experience compared to men[39]. While the primary analyses examined sexual minority subgroups in relation to the completely heterosexual group, some striking patterns emerged comparing sexual minority subgroups to one another. For example, bisexual women were the most likely to have been uninsured (13.8%) compared to completely heterosexual (3.9%) women as well as compared to mostly heterosexual (5.7%) and lesbian (4.4%) women; *p*-value <0.001. These findings align with the literature documenting bisexuals often experience some of the highest burdens of adverse health, even compared to other sexual minority subgroups[40].

The GUTS cohort is made up of children of NHS2 participants, so results in this sample may not generalize to other populations; this cohort is predominantly White race/ethnicity, their mothers are all nurses, and the majority of participant's annual household income during their childhood was ≥$75,000. Given this high social status, our estimates may underestimate the prevalence of unemployment, a lack of health insurance, a lack of healthcare access, and poor HRQL. However, the fact that these disparities are pervasive in a cohort that predominantly holds high social status is striking. While our focus was on employment, health insurance, and healthcare access, there may be other factors that mediate the sexual orientation-related HRQL disparities including bullying victimization, social status, and others[14]. The lack of explanation in these disparities by HRQL in this cohort may be due to the participants' young age. Future research should explore how employment and healthcare may interact with age to drive worse HRQL as people age. Data were cross-sectional and limited on some of our variables such as health insurance and healthcare access. Future longitudinal studies could explore more detailed types of health insurance coverage as well as other measurements of healthcare access.

This study has a number of strengths including the large sample drawn from young adults living across the United States. Building off the recently published data of sexual orientation-related disparities of HRQL index scores[14], this is the first study to examine these disparities across HRQL domains and sexual minority subgroup while also evaluating the role of additional factors including employment, health insurance, and healthcare access. Utilizing the EQ-5D-5L to measure HRQL allows the findings to be incorporated into cost-effectiveness research, which can inform public policy decisions.

These sexual orientation-related disparities in employment and health insurance in a population with high social status highlight the ubiquity of sexual orientation inequities in the employment and healthcare systems. The U.S. Supreme Court's recent expansion of marriage rights to adults nationwide in same-sex relationships should lessen some of the sexual orientation-related disparities in health insurance. However, the adverse effects of previous bans are likely to persist[41,42]. Additionally, 28 states across the U.S. currently have no employment non-discrimination law covering sexual orientation—3 of these states have laws preventing the passage or enforcement of local non-discrimination laws[20]. Until all people,

For peer review only - http://bmjopen.bmj.com/site/about/guidelines.xhtml

regardless of sexual orientation, are treated equally in the eyes of the law including with non-discrimination laws protecting employment as well as housing, public accommodations, and credit/lending, sexual orientation-related health disparities will persist.

Contributorship: Dr. Charlton conceptualized the project, supervised the analyses, and led the development and writing of the article. Dr. Austin supervised data collection and, along with Drs. Gordon, Reisner, and Samnaliev, aided in the interpretation of data and critically reviewed the manuscript for important intellectual content. Mr. Sarda conducted the analyses.

Funding: Dr. Charlton was supported by grant F32HD084000 and Dr. Austin by R01HD057368 and R01HD066963 from the *Eunice Kennedy Shriver* National Institute of Child Health and Human Development, National Institutes of Health. Additional funds were provided to Dr. Charlton by GLMA: Health Professionals Advancing LGBT Equality's Lesbian Health Fund and by MRSG CPHPS 130006 from the American Cancer Society. Dr. Reisner was partly supported by grant CER-1403-12625 from the Patient-Centered Outcomes Research Institute. Additional funds were provided to Dr. Charlton by the American Cancer Society through grant number MRSG CPHPS 130006. Additionally, an abstract of this work was presented at the Society for Adolescent Health and Medicine Annual Meeting and GLMA Annual Conference on LGBT Health.

Competing Interests: None declared.

Ethics Approval: This study was approved by the Brigham and Women's Hospital Institutional Review Board.

Data Sharing: No additional data available.

References

1 Sears B, Mallory C. Documented evidence of employment discrimination and its effects on LGBT people. The Williams Institute 2011.
2 Miller W, Robinson LA, Lawrence RS, editors. Valuing health for regulatory cost-effectiveness analysis. Washington, D.C.: : National Academies Press 2006. doi:10.17226/11534
3 Badgett MVL, Ash MA. Separate and unequal: the effect of unequal access to employment-based health insurance on gay, lesbian, and bisexual people. *Contemp Econ Policy* 2006;**24**:582–99.
4 Austin EL, Irwin JA. Health behaviors and health care utilization of southern lesbians. *Womens Heal Issues* 2010;**20**:178–84. doi:10.1016/j.whi.2010.01.002
5 Badgett MVL, Schneebaum A. The impact of wage equality on sexual orientation poverty gaps. The Williams Institute; 2015.
6 Badgett MVL, Durso LE, Schneebaum A. New patterns of poverty in the lesbian, gay, and bisexual community. The Williams Institute; 2013.
7 Buchmueller T, Carpenter CS. Disparities in Health Insurance Coverage, Access, and Outcomes for Individuals in Same-Sex Versus Different-Sex Relationships, 2000?2007. *Am J Public Health* 2010;**100**:489–95. doi:10.2105/AJPH.2009.160804
8 Diamant AL, Wold C, Spritzer K, *et al.* Health behaviors, health status, and access to and use of health care: a population-based study of lesbian, bisexual, and heterosexual women. *Arch Fam Med* 2000;**9**:1043–51.
9 Gonzales G, Blewett LA. National and state-specific health insurance disparities for adults in same-sex relationships. *Am J Public Health* 2014;**104**:e95–104.

For peer review only - http://bmjopen.bmj.com/site/about/guidelines.xhtml

doi:10.2105/AJPH.2013.301577
10 Gonzales G, Henning-Smith C. The affordable care act and health insurance coverage for lesbian, gay, and bisexual adults: analysis of the behavioral risk factor surveillance system. *LGBT Heal* 2017;**4**:62–7. doi:10.1089/lgbt.2016.0023
11 Heck JE, Sell RL, Gorin SS. Health care access among individuals involved in same-sex relationships. *Am J Public Health* 2006;**96**:1111–8. doi:10.2105/AJPH.2005.062661
12 Pals H, Waren W. Winners and losers in health insurance: access and type of coverage for women in same-sex and opposite-sex partnerships. *Women Health* 2014;**54**:94–114. doi:10.1080/03630242.2013.870633
13 Prokos AH, Keene JR. Poverty among cohabiting gay and lesbian, and married and cohabiting heterosexual families. *J Fam Issues* 2010;**31**:934–59. doi:10.1177/0192513X09360176
14 Austin SB, Gordon AR, Ziyadeh NJ, *et al.* Stigma and health-related quality of life in sexual minorities. *Am J Prev Med* 2017;**57**:[Epub ahead of print] 566-587.
15 Case P, Austin SB, Hunter DJ, *et al.* Sexual orientation, health risk factors, and physical functioning in the Nurses' Health Study II. *J Womens Heal* 2004;**13**:1033–47. doi:10.1089/jwh.2004.13.1033
16 Cochran SD, Mays VM. Physical health complaints among lesbians, gay men, and bisexual and homosexually experienced heterosexual individuals: results from the California Quality of Life Survey. *Am J Public Health* 2007;**97**:2048–55. doi:10.2105/AJPH.2006.087254
17 Eriksson LE, Berglund T. Health-related quality of life in homo- and bisexual men attending a sexually transmitted disease clinic in Sweden. *Int J STD AIDS* 2007;**18**:207–11. doi:10.1258/095646207780132442
18 Boladale M, Olakunle O, Olutayo A, *et al.* Sexual orientation and quality of life among students of Obafemi Awolowo University (OAU), Nigeria. *Afr Health Sci* 2015;**15**:1065–73. doi:10.4314/ahs.v15i4.3
19 Traeen B, Martinussen M, Vittersø J, *et al.* Sexual orientation and quality of life among university students from Cuba, Norway, India, and South Africa. *J Homosex* 2009;**56**:655–69. doi:10.1080/00918360903005311
20 Movement Advancement Project. Non-Discrimination Laws. 2017.http://www.lgbtmap.org/equality-maps/non_discrimination_laws (accessed 28 Jun 2017).
21 Remafedi G, Resnick M, Blum R, *et al.* Demography of sexual orientation in adolescents. *Pediatrics* 1992;**89**:714–21.
22 Johnson JA, Coons SJ. Comparison of the EQ-5D and SF-12 in an adult US sample. *Qual Life Res* 1998;**7**:155–66.
23 EuroQol Group. EQ-5D-5L. 2017.https://euroqol.org/eq-5d-instruments/eq-5d-5l-about/ (accessed 12 Jan 2017).
24 Gordon AR, Krieger N, Okechukwu CA, *et al.* Decrements in health-related quality of life associated with gender nonconformity among U.S. adolescents and young adults. Qual. Life Res. 2017;:1–10. doi:10.1007/s11136-017-1545-1
25 Agency for Healthcare Research and Quality. U.S. valuation of the EuroQol EQ-5 health states: research initiative in clinical economics. 2012.http://www.ahrq.gov/professionals/clinicians-providers/resources/rice/EQ5Dproj.html (accessed 12 Jan 2017).
26 van Hout B, Janssen MF, Feng Y-S, *et al.* Interim scoring for the EQ-5D-5L: mapping the EQ-5D-5L to EQ-5D-3L value sets. *Value Heal* 2012;**15**:708–15. doi:10.1016/j.jval.2012.02.008
27 Lubetkin EI, Jia H, Franks P, *et al.* Relationship among sociodemographic factors, clinical conditions, and health-related quality of life: examining the EQ-5D in the u.s. general

For peer review only

population. *Qual Life Res* 2005;**14**:2187–96. doi:10.1007/s11136-005-8028-5
28 Goldsmith KA, Dyer MT, Schofield PM, *et al.* Relationship between the EQ-5D index and measures of clinical outcomes in selected studies of cardiovascular interventions. *Health Qual Life Outcomes* 2009;**7**:96. doi:10.1186/1477-7525-7-96
29 Pullenayegum EM, Tarride J-E, Xie F, *et al.* Analysis of health utility data when some subjects attain the upper bound of 1: are Tobit and CLAD models appropriate? *Value Heal* 2010;**13**:487–94. doi:10.1111/j.1524-4733.2010.00695.x
30 Bilger M, Finkelstein EA, Kruger E, *et al.* The effect of weight loss on health, productivity, and medical expenditures among overweight employees. *Med Care* 2013;**51**:471–7. doi:10.1097/MLR.0b013e318286e437
31 Pavot W, Diener E. Review of the satisfaction with life scale. *Psychol Assess* 1993;**5**:164–72. doi:10.1007/978-90-481-2354-4
32 Lyubomirsky S, Lepper HS. A measure of subjective happiness: preliminary reliability and construct validation. *Soc Indic Res* 1999;**46**:137–55. doi:10.1023/A:1006824100041
33 The World Health Organization Quality of Life Assessment (WHOQOL): development and general psychometric properties. *Soc Sci Med* 1998;**46**:1569–85.
34 Ware J, Kosinski M, Keller SD. A 12-item short-form health survey: construction of scales and preliminary tests of reliability and validity. *Med Care* 1996;**34**:220–33.
35 Ware J. *SF-36 physical and mental health summary scales: a user's manual*. Boston: : Health Institute New England Medical Center 1994.
36 Fredriksen-Goldsen KI, Kim H-J, Barkan SE, *et al.* Disparities in health-related quality of life: a comparison of lesbians and bisexual women. *Am J Public Health* 2010;**100**:2255–61. doi:10.2105/AJPH.2009.177329
37 Marti-Pastor M, Perez G, German D, *et al.* Health-related quality of life inequalities by sexual orientation: Results from the Barcelona Health Interview Survey. *PLoS One* 2018;**13**:e0191334. doi:10.1371/journal.pone.0191334
38 Galdas PM, Cheater F, Marshall P. Men and health help-seeking behaviour: literature review. *J Adv Nurs* 2005;**49**:616–23. doi:10.1111/j.1365-2648.2004.03331.x
39 Fillingim RB, King CD, Ribeiro-Dasilva MC, *et al.* Sex, gender, and pain: a review of recent clinical and experimental findings. *J pain Off J Am Pain Soc* 2009;**10**:447–85. doi:10.1016/j.jpain.2008.12.001
40 Gorman BK, Denney JT, Dowdy H, *et al.* A New Piece of the Puzzle: Sexual Orientation, Gender, and Physical Health Status. *Demography* 2015;**52**:1357–82. doi:10.1007/s13524-015-0406-1
41 Hatzenbuehler ML, McLaughlin KA, Keyes KM, *et al.* The Impact of Institutional Discrimination on Psychiatric Disorders in Lesbian, Gay, and Bisexual Populations: A Prospective Study. *Am J Public Health* 2010;**100**:452–9. doi:10.2105/AJPH.2009.168815
42 Hatzenbuehler ML, O'Cleirigh C, Grasso C, *et al.* Effect of Same-Sex Marriage Laws on Health Care Use and Expenditures in Sexual Minority Men: A Quasi-Natural Experiment. *Am J Public Health* 2012;**102**:285–91. doi:10.2105/AJPH.2011.300382

For peer review only - http://bmjopen.bmj.com/site/about/guidelines.xhtml

STROBE Statement—Checklist of items that should be included in reports of ***cohort studies***

	Item No	Recommendation	Page
**Title and abstract**	1	(*a*) Indicate the study's design with a commonly used term in the title or the abstract	1
		(*b*) Provide in the abstract an informative and balanced summary of what was done and what was found	2
**Introduction**			
Background/rationale	2	Explain the scientific background and rationale for the investigation being reported	3
Objectives	3	State specific objectives, including any prespecified hypotheses	3
**Methods**			
Study design	4	Present key elements of study design early in the paper	3-5
Setting	5	Describe the setting, locations, and relevant dates, including periods of recruitment, exposure, follow-up, and data collection	3-5
Participants	6	(*a*) Give the eligibility criteria, and the sources and methods of selection of participants. Describe methods of follow-up	3-5
		(*b*) For matched studies, give matching criteria and number of exposed and unexposed	N/A
Variables	7	Clearly define all outcomes, exposures, predictors, potential confounders, and effect modifiers. Give diagnostic criteria, if applicable	3-5
Data sources/ measurement	8*	For each variable of interest, give sources of data and details of methods of assessment (measurement). Describe comparability of assessment methods if there is more than one group	3-5
Bias	9	Describe any efforts to address potential sources of bias	3-5
Study size	10	Explain how the study size was arrived at	3
Quantitative variables	11	Explain how quantitative variables were handled in the analyses. If applicable, describe which groupings were chosen and why	3-5
Statistical methods	12	(*a*) Describe all statistical methods, including those used to control for confounding	3-5
		(*b*) Describe any methods used to examine subgroups and interactions	3-5
		(*c*) Explain how missing data were addressed	3-5
		(*d*) If applicable, explain how loss to follow-up was addressed	3
		(*e*) Describe any sensitivity analyses	N/A
**Results**			
Participants	13*	(a) Report numbers of individuals at each stage of study—eg numbers potentially eligible, examined for eligibility, confirmed eligible, included in the study, completing follow-up, and analysed	5-9
		(b) Give reasons for non-participation at each stage	5-9
		(c) Consider use of a flow diagram	N/A
Descriptive data	14*	(a) Give characteristics of study participants (eg demographic, clinical, social) and information on exposures and potential confounders	5-6
		(b) Indicate number of participants with missing data for each variable of interest	4-5
		(c) Summarise follow-up time (eg, average and total amount)	3-5
Outcome data	15*	Report numbers of outcome events or summary measures over time	5-9
Main results	16	(*a*) Give unadjusted estimates and, if applicable, confounder-adjusted estimates and their precision (eg, 95% confidence interval). Make clear	5-9

For peer review only

		which confounders were adjusted for and why they were included	
		(*b*) Report category boundaries when continuous variables were categorized	6-10
		(*c*) If relevant, consider translating estimates of relative risk into absolute risk for a meaningful time period	N/A
Other analyses	17	Report other analyses done—eg analyses of subgroups and interactions, and sensitivity analyses	N/A
**Discussion**			
Key results	18	Summarise key results with reference to study objectives	10
Limitations	19	Discuss limitations of the study, taking into account sources of potential bias or imprecision. Discuss both direction and magnitude of any potential bias	11
Interpretation	20	Give a cautious overall interpretation of results considering objectives, limitations, multiplicity of analyses, results from similar studies, and other relevant evidence	11-12
Generalisability	21	Discuss the generalisability (external validity) of the study results	11-12
**Other information**			
Funding	22	Give the source of funding and the role of the funders for the present study and, if applicable, for the original study on which the present article is based	12

*Give information separately for exposed and unexposed groups.

Note: An Explanation and Elaboration article discusses each checklist item and gives methodological background and published examples of transparent reporting. The STROBE checklist is best used in conjunction with this article (freely available on the Web sites of PLoS Medicine at http://www.plosmedicine.org/, Annals of Internal Medicine at http://www.annals.org/, and Epidemiology at http://www.epidem.com/). Information on the STROBE Initiative is available at http://www.strobe-statement.org.

For peer review only

For peer review only - http://bmjopen.bmj.com/site/about/guidelines.xhtml
